# Clinical Characteristics and Surgical Problems of Ruptured Globe Injury^☆^

**DOI:** 10.1016/j.curtheres.2012.10.002

**Published:** 2013-06

**Authors:** Hongsheng Bi, Yan Cui, Yang Li, Xingrong Wang, Jianhua Zhang

**Affiliations:** Shierming Eye Hospital of Shandong Province, Affiliated Eye Hospital of Shandong University of Traditional Chinese Medicine, Jinan, China

**Keywords:** choroidal lesion, ruptured globe injury, trauma

## Abstract

**Background:**

Ocular trauma is a major cause of vision loss, especially in the young patients, and is the leading cause of unilateral blind in China.

**Objective:**

The aims of this report are to analyze ciliary and choroidal lesion characteristics and outcomes of a group of patients with ruptured globe injuries and discuss finding a more effective treatment protocol. Here we report our experience treating ruptured globe injuries.

**Methods:**

Seventy-five patients (75 eyes) with a diagnosis of ruptured globe injuries were selected from 264 patients with open globe injuries at the Shierming Eye Hospital of Shandong Province between January 2009 and December 2011. General information and clinical characteristics such as ciliary and choroidal lesion features were reviewed.

**Results:**

Of the 75 patients, 85.3% were men, and the average age of the patients was 37.2 years (range, 6–63 years). The right eye was injured in 52.0%; enucleation was performed in 9 patients. There was no light perception, in the final corrected visual acuity in another 3 patients. The ratio of better visual acuity (better than 0.1) increased from 0 preoperatively to 16.0% postoperatively. Among the 75 patients with ruptured globe injuries, 13 had ciliary injury and 47 (62.7%) had choroidal injuries. Both ciliary and choroidal injuries were detected in 15 patients. Retinal tissue incarceration during sclera suturing was usually the vital point leading to unfavorable results.

**Conclusions:**

Ruptured globe injury usually results in severe visual acuity damage. Active treatment could help to restore visual acuity in patients to some degree. Some effective treatment protocols for ruptured globe injuries could be followed. Some unsuitable procedures in primary treatment should be avoided to achieve a better prognosis.

## Introduction

Ocular trauma is a very important cause of visual damage, especially in young patients.^1^ Trauma can destroy the normal structure of the eyeball directly; as a result, ocular physiology function may also be affected. In the United States, eye injuries cost >$300 million per year due to lost productivity, medical expenses, and workers’ compensation.^2^ In China, ocular trauma is the primary cause of unilateral blindness. According to the Birmingham Eye Trauma Terminology (BETT) criteria,^3,4^ rupture is defined as a full-thickness wound of the eye wall caused by a blunt object. In this study, the preoperative visual acuity of patients with severe ruptured globe injuries was poor mostly. After routine surgery, patients’ visual acuity improved to varying degrees. Sometimes the visual acuity prognosis of these patients depended on whether they received suitable primary treatment. Therefore, we report here our experience in treating severe ruptured globe injuries, which may be helpful for junior oculists.

We reviewed retrospectively the clinical data of 75 patients with severe ruptured globe injuries from 264 open globe injuries treated in the Shierming Eye Hospital of Shandong Province from January 2009 to December 2011. Here we describe the features and surgical interventions and try to determine more effective ways to treat severe ruptured globe injuries.

## Methods

### Clinical Data

A retrospective chart review was conducted on 75 patients (75 eyes) treated in the Shierming Eye Hospital of Shandong Province of 264 patients with open globe injuries between January 2009 and December 2011. According to the BETT criteria,^3^ 75 patients (28.4%) had a diagnosis of ruptured globe injury. The data recorded included age, sex, cause of trauma, preoperative and postoperative visual acuity, preoperative and postoperative intraocular pressure (IOP), special examinations (ie, B-scan, ultrasound biomicroscopy, and optical coherence tomograph), and surgical method. The follow-up was 12 to 36 months after surgical intervention.

In this study, we detected ciliary injury by using an ultrasound biomicroscope, (Meda Co., Ltd., Tianjin, China). Ciliary injury was defined as any injury affecting the ciliary body, including ciliary body detachment, ciliary body defect, ciliary process atrophy, and formation of a ciliary membrane. Choroidal injury included choroidal laceration, choroidal detachment, choroidal rupture, choroidal incarceration, and choroidal defect.

### Treatment Protocol

On arrival at our hospital, detailed case histories are recorded. The protocol is as follows. For the open wound and tissue prolapse, we try to recover normal global anatomic structure with watertight closure. For iris, choroid, and retinal prolapse, the tissue should be rinsed and repositioned; the prolapsed vitreous body is removed.

A complete ophthalmic examination is performed to evaluate whether there is an intraocular foreign body by B ultrasonic scanning, ultrasound biomicroscopy, and computed tomography. If there is a metal intraocular foreign body and infection is suspected, the surgery is performed as soon as possible. Determination of the incision site and pathway, such as a previous wound, corneal scleral incision, and pars plana incision, is made according to the foreign body feature, location, and disease characteristics.

After suturing the corneal and scleral laceration, a second surgery is performed 2 weeks later. For some patients with anterior chamber hyphema, anterior chamber irrigation after injection of viscoelastics could prevent blood staining of the cornea. A complete vitrectomy is performed via a pars plana incision, avoiding the injury site.^5^ If there is ciliary body injury, detachment, or hyphema, it is difficult to determine the injection tube location. The incision site can be moved near the limbus. Retinomy or retinoectomy may be preferred according to the trauma status. The lens is tried to see whether it is transparent enough. Otherwise lensectomy or phacoemulsification is performed first.

Silicone oil is removed 3 to 6 months postoperatively. Artificial lens implantation is performed in patients with good corrected visual acuity. In some patients with pathological proliferation changes, the membrane should be peeled off or retinoectomy attempted combined with refilling of silicone oil for retinal reattachment.

## Results

### General Data

Of the 75 patients, 64 (85.3%) were male. The average age was 37.2 years (range, 6–63 years). The right eye was injured in 52.0% of patients, and no patient in this series had bilateral injuries. The majority of the patients (81.3%) performed physical labor. Nine of the 75 patients (12.0%) were children 6 to 15 years of age. The primary cause of rupture was accidental objects (ie, being unexpectedly struck), accounting for 82.7%, such as a chair, abrasive wheel, concrete iron, stone, and bicycle handlebars. In 9.3% (7 of 75) of explosive injuries, the main cause was fireworks and detonators, which accounted for 6.7% and 2.6%, respectively. Foreign bodies were detected in 5 of 7 explosive injuries. In addition, 2 patients were hurt by being hit with a fist, 3 were hurt in a traffic accident, and 1 was hurt after drinking (Table I), due to slipping and falling. Among the 75 patients with rupture injuries, 13 (17.3%) had ciliary injury, 47 (62.7%) had choroidal injuries, and ciliary and choroidal injuries were both detected in 15 patients. Enucleation was performed in 2 patients with blood staining of the cornea and severe intraocular tissue defect; light perception or better was achieved in an additional 8 patients with hyphema. A closed funnel-like detached retina was confirmed during exploratory surgery in 10 patients with retinal tissue incarceration during sclera suturing, and these patients had a final visual acuity of finger counting or worse.

### Visual Acuity Prognosis

**Figure 1:**
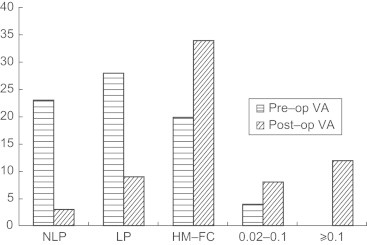
Comparison of preoperative (Pre-op) visual acuity (VA) and postoperative (Post-op) VA. In addition, enucleation was performed in 9 patients. FC = finger counting; HM = hand motion; NLP = no light perception.

On initial presentation, the visual acuity of all patients was <0.1. In 23 patients (30.7%) with no light perception, 13 patients were injured by objects such as wood, stone, or a high-speed grinding wheel. Seven patients were injured in an explosion, 2 patients by being hit with a fist, and 1 was hurt by the bicycle handlebars. Finally, besides 9 patients who underwent enucleation, 3 additional patients had final visual acuity of no light perception; the remaining 11 patients had light perception or better (Figure 1).

After surgery, the best corrected visual acuity on the last recorded follow-up of 12 patients (16%) was ≥0.1. It ranged from 0.02 to 0.1 in 8 patients (10.7%). We also performed a study of the differences in visual acuity after treatment among those with ciliary injury, choroidal injury, and both (Table II). The results showed that the prognosis of ciliary injury was much better than in the other 2 groups (α = 0.05, χ^2^ = 57.817 [χ^2^ tests], *P* < 0.001).

### Choroid Injuries

**Figure 2:**
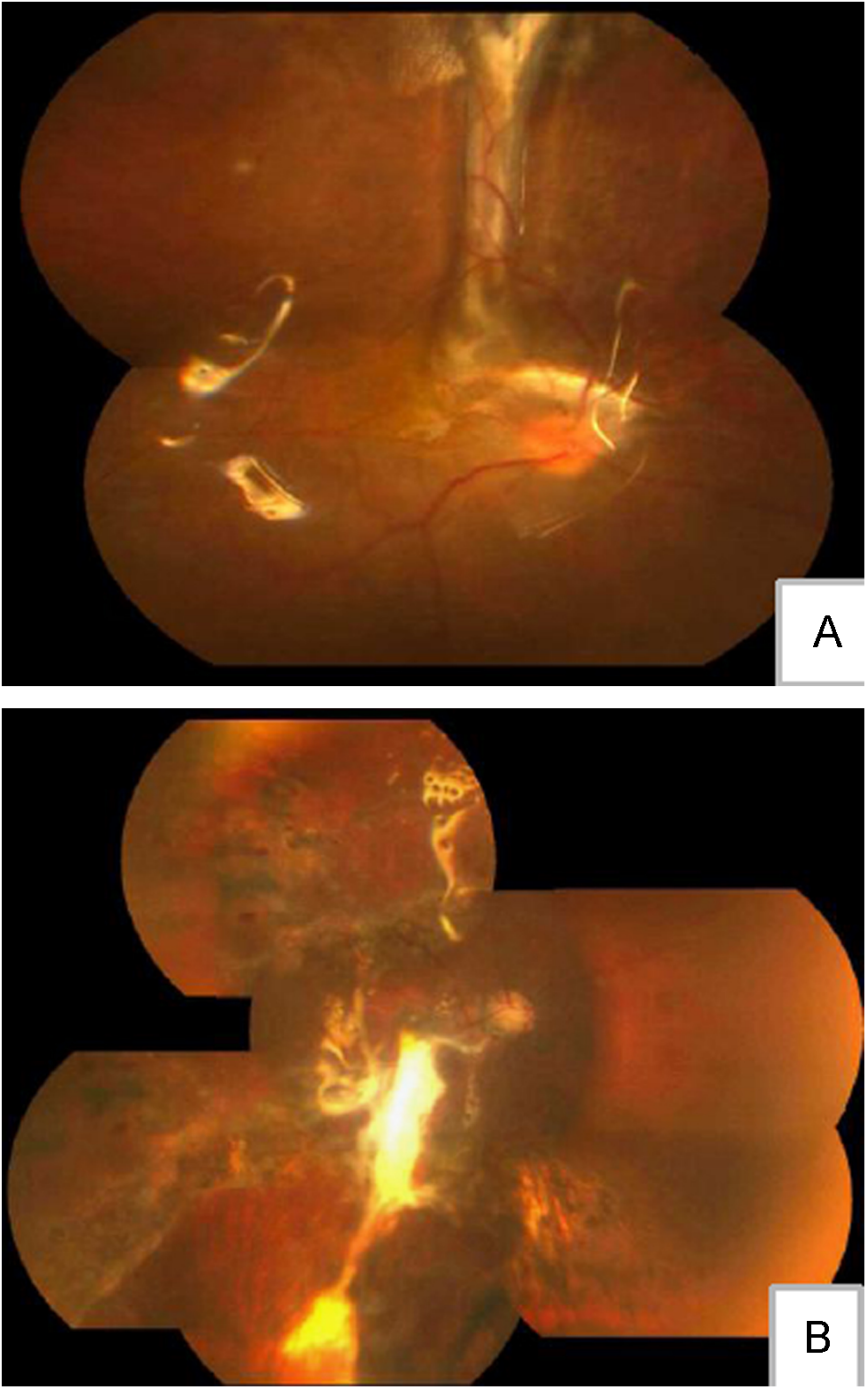
Because of the tissue characteristics of the choroid, it is difficult to stretch and smooth out the choroid. Until now there has been no effective way to repair a choroidal injury. Choroidal rupture could result in choroidal scar proliferation as local prominence (A). Choroidal rupture at the fovea could result in devastating visual consequences (B).

Most of the patients had choroidal injuries such as choroidal laceration and defect. Because of the characteristics of choroidal tissue, it is difficult to stretch and smooth it out. Until now, there was no effective way to repair choroidal injury. Some choroidal lesions (eg, choroidal rupture at the fovea) could result in devastating visual consequences (Figure 2). In this study, only 3 patients with choroidal injuries preoperatively had visual acuity of ≥0.1 postoperatively.

**Figure 3:**
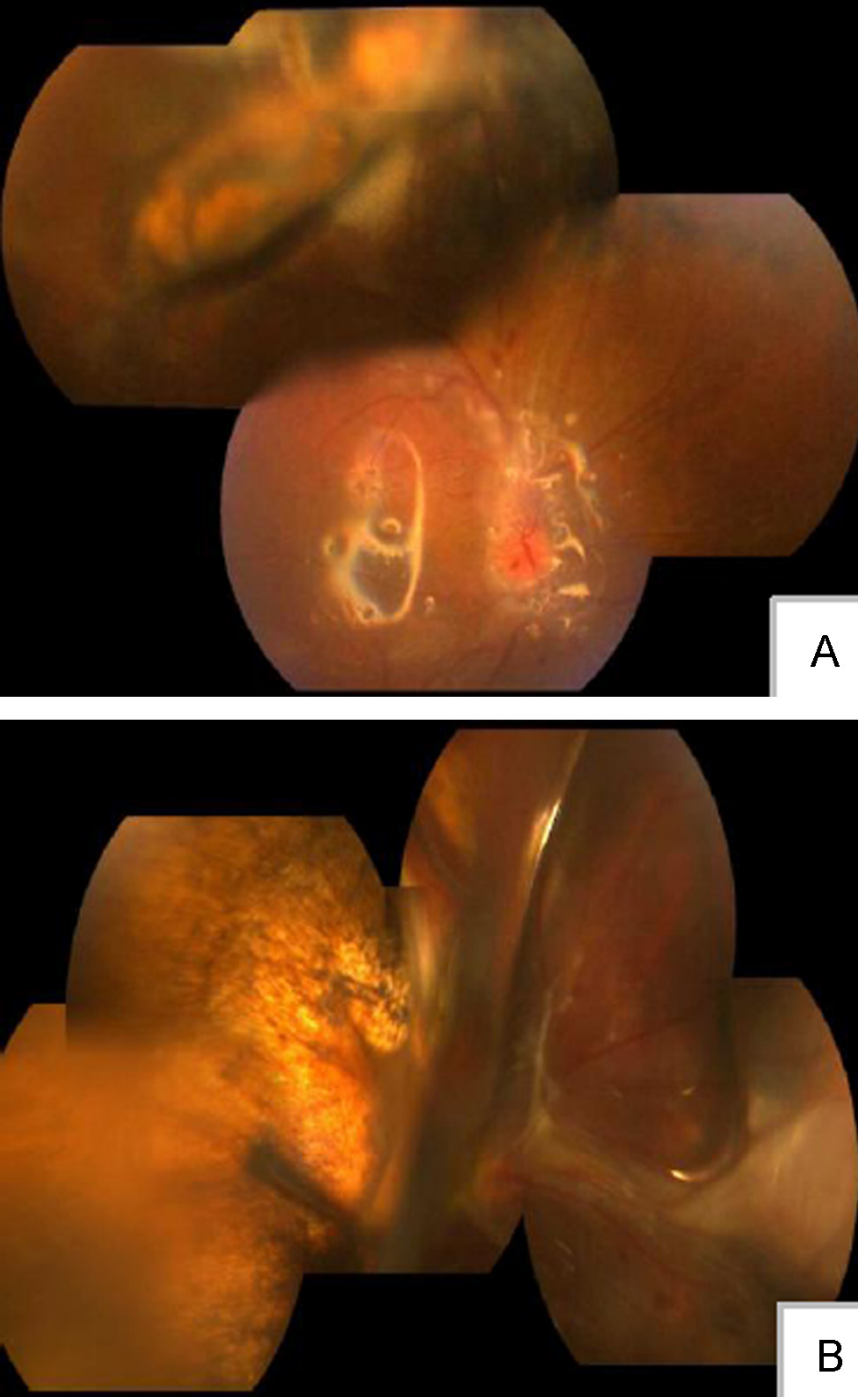
Some patients with local choroidal prominence were misdiagnosed as choroidal detachment, in fact, the prominence was caused by uneven or crimpled ruptured choroidal tissue. The best corrected visual acuity of this patient was 0.3 after silicone oil removal surgery (A). For some choroidal defect patients, the naked sclera could be identified by the light guide as a “lantern phenomenon.” The retina is attached to the choroid, but choroid could be separated from the sclera. The patient could retain some visual function in some quadrants, but this patient's eye was silicone oil dependent (B).

For some patients with a choroidal defect, naked sclera could be identified by the light guide as a “lantern phenomenon.” After surgery, the retina was attached to the choroid, but the choroid could be separated from the sclera. The patients could retain some visual function in some quadrants (Figure 3).

### Postoperative Complications and Multiple-Operation Problem

**Figure 4:**
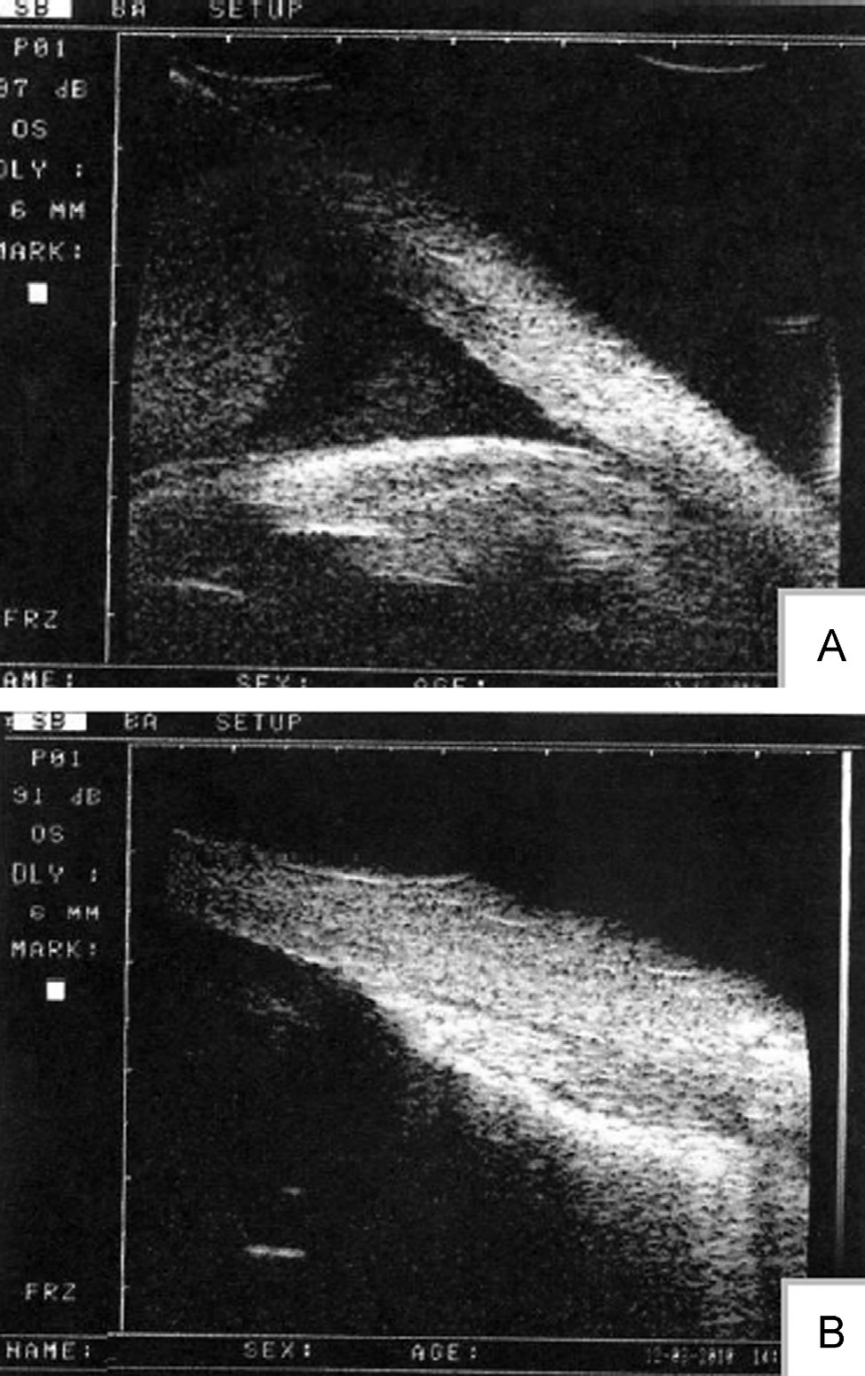
In many severely injured eyes, blood flow to the ciliary body is compromised by damage to the long posterior and anterior ciliary arteries and the major arterial circle. As a result, hypotony, iris atrophy, and paralytic mydriasis are common complications. The iris was present at the first presentation of patient (A); 3 months later, the iris had disappeared (B).

There were some complications postoperatively such as secondary cataract (5.3%, n = 4), belted corneal degeneration (2.7%, n = 2), and iris atrophy or irregular pupil (9.3%, n = 7) (Figure 4). Fortunately, there were no patients with a diagnosis of sympathetic ophthalmia.

**Figure 5:**
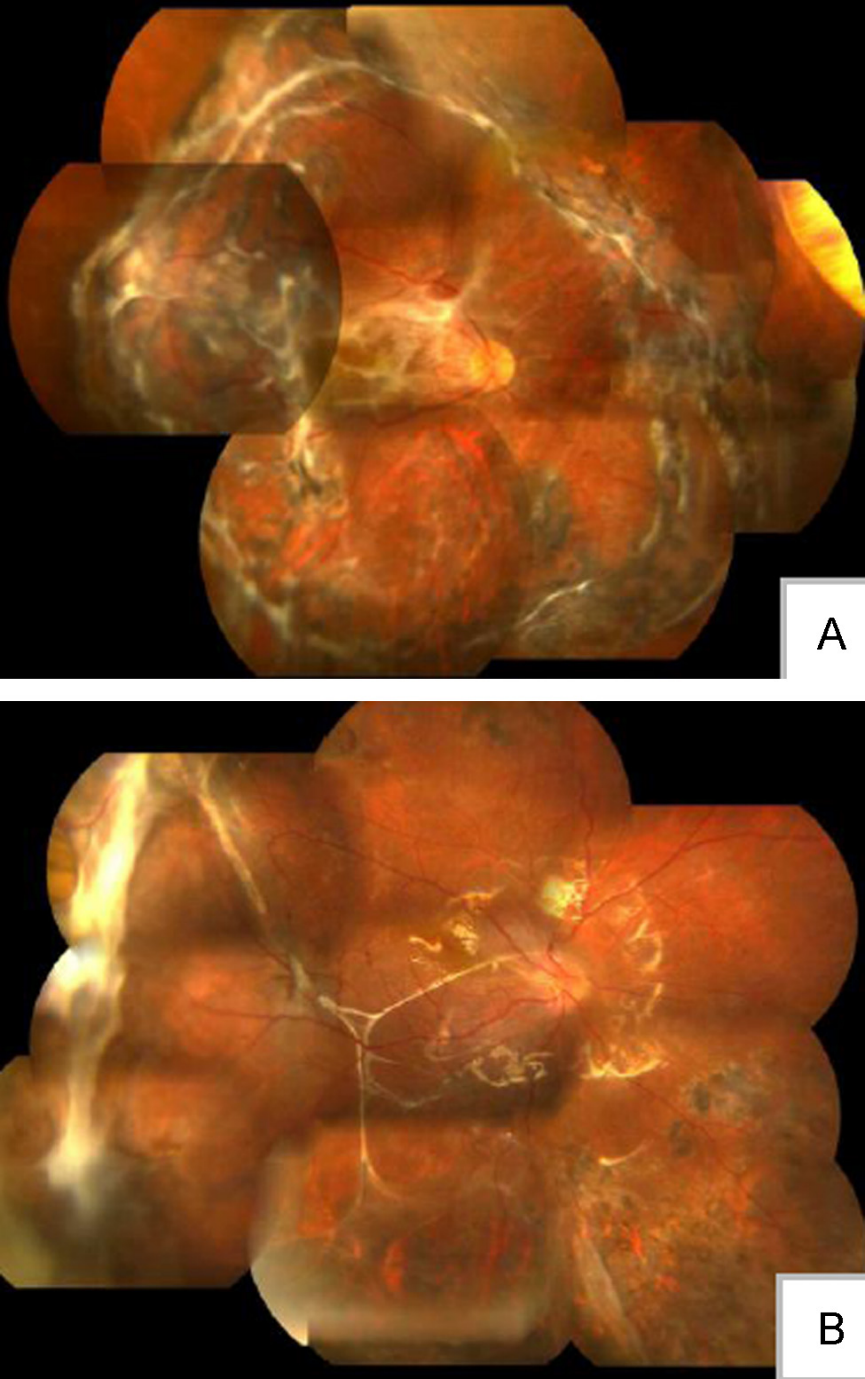
During the second surgery, proliferation is the key problem needing to be resolved. For ruptured globe trauma, silicone oil tamponade could alleviate the proliferative reaction. Proliferation is usually focused in the macular area because of gravity. Patients could feel vision distortion or blurred vision. If proliferation occurs, peeling the membrane could be performed 3 to 4 months after the first vitrectomy. Most of the patients with silicone oil tamponade during the second surgery could stop the proliferative reaction and keep the retina attached (A); however, there is no good treatment method for subretinal membrane peeling (B).

Of 55 patients who underwent >2 surgeries, silicone oil was removed in 39 patients. Nine patients received an intraocular lens implant. Silicone oil was refilled in 16 patients because of lower IOP (<8 mm Hg) or proliferation response. During the second surgery, proliferation was the key problem that needed to be resolved (Figure 5).

## Discussion

Our hospital is a central eye hospital in Shandong Province serving the whole area. Therefore, this study reflected the current status of the diagnosis and treatment of ruptured globe injury in the Shandong provincial district.

Ocular trauma is a common disease that can result in severe visual function damage.^6^ It is the primary cause of unilateral blindness in China. An unsuitable primary surgical intervention often results in poor visual prognosis. Hence, we have the following suggestions for the primary surgery: (1) The prolapsed iris, choroid, and retina should be repositioned after complete irrigation, and the prolapsed vitreous body can be removed. Otherwise, retinal incarceration and closed tunnel detachment will result in poor visual acuity and severe proliferative response. (2) The ends of the ocular rupture wound must be determined carefully and sutured completely to keep the IOP normal. (3) For some patients with anterior chamber hyphema, anterior chamber irrigation after tamponade of viscoelastics could prevent blood staining of the cornea.

From the clinical data, the average age of patients with severe ruptured globe injury was 37.2 years old. The patients were young and able to work. There were more male than female patients.^7,8^ Some injuries occurred when the patients were at work where there were no safety measures in place. The majority of the laborers had no eye protection, which was a major cause of injury.^9^ Only 32% of adults have ocular protection while performing visually threatening labor in the United States.^10^ Children are another group easily injured because of a lack of safety knowledge and less sense of self-protection. The visual impact after ruptured globe injuries is usually severe. Therefore, it is very important to teach this group ocular protection.

Explosive injury is usually combined with a foreign body; in the present series, foreign bodies were detected in 5 of 7 explosive injuries. Explosive injury is mostly caused by fireworks during the Chinese New Year festival. According to the World Health Organization, childhood blindness is one of the major types of avoidable blindness.^11^ Therefore, it is important to teach safety, especially to children.^12^

### Postoperative Visual Acuity

A rupture injury lesion is large and irregular and easily results in ocular contents prolapse and visual acuity damage.^13^ For the patients with poor visual acuity (ie, less than hand motion even without light perception), their visual acuity could be improved after active, effective treatment, such as surgery, be promptly carried out. Visual acuity improved in approximately half of patients. There were several reasons that patients had no light perception preoperatively. First, severe hemorrhage from the ciliary body or choroid during trauma could shelter the macula. Second, severe globe rupture caused the incarcerated retina to lesion. The residual retina was detached and funnel-like and hid the macula. For the hemorrhage patients, removing the bleeding with vitrectomy, patients could get better visual acuity. In patients with a funnel-like detached retina, remember not to dissect the incarcerated retina first. Remove the vitreous inside the funnel and peel off the subretinal membrane before dissection of the peripheral retina. After reattaching the retina by gas or perfluorocarbon liquid, we photocoagulate the peripheral retina and attempt to retain visual acuity.^14^ On the other hand, if the peripheral incarcerated retina is dissected first, the vitreous body will stick to the residual retina and be difficult to separate. In some cases, a funnel-like detached retina is difficult to reattach. We think that there are 2 reasons for this. First, commotio retinal tissue during trauma releasing some inflammatory cytokines or some active materials results in retinal tissue melting. Second, in patients with subretinal hemorrhage, plasmin in the blood results in retinal degeneration and adherence.

### Treatment of Choroid Injuries

Ruptured globe injury is usually combined with choroidal tissue lesions; however, there is no way to suture a choroidal rupture. At present, what we can do is to suture a sclera rupture and keep choroidal rupture alignment. In some patients with a choroidal defect caused by trauma, try to attach the retina to the choroid. Partial visual acuity could be retained in some patients whose choroid could not be attached to the sclera.

Choroidal injury usually results in severe scarring and proliferation.^15,16^ The retina and vitreous body trapped inside the lesion should be removed and released. Adhesion between the choroidal lesion and scar should be released. Otherwise, scarring condensation during healing could pull the retina, especially with scarring near the macula. According to the kinetic action of scarring, perform a retinoectomy to align Henle fiber to ensure normal macular structure.

The iris blood supply is from the major arterial circle inside the ciliary body.^17,18^ The iris arterial circle is easily damaged during the open globe injury; as a result, iris atrophy or paralytic mydriasis is a common complication. For endophthalmitis patients, the iris is affected by bacterial endotoxin. Obstruction of the root excision in a tonic iris could induce dislocation of the silicone oil into the anterior chamber. The iris root could be dissected with paracentesis of the anterior chamber followed by miosis. However, the iris is not sensitive to both miosis and mydriasis.

### Multiple-Operation Problem

There were 55 patients who underwent multiple operations. What is more, there were 16 patients in whom silicone oil was refilled during the third or fourth surgery. For these 16 patients, severe proliferation occurred at the foreign-body incarceration sites in 5 patients. Therefore, this suggests that we should try to separate the retina from the trauma lesion site and follow with preventive coagulation around the lesion. Complete vitrectomy is less prone to result in fibrous proliferation than partial vitrectomy.^19^ Therefore, it is important to perform a complete vitrectomy including the base vitreous around the ciliary body. Try to keep more retinal tissue and prevent pigment cells from floating out. For ruptured globe trauma, silicone oil tamponade could alleviate the proliferative reaction.^20^ The proliferation is usually focused in the macular area because of gravity. Patients could feel vision distortion or blurred vision. If proliferation occurs, peeling off the membrane could be performed 3 to 4 months after the first vitrectomy. In most of the patients with silicone oil tamponade during the second surgery, the proliferative reaction could stop and the retina could remain attached.^21^

### Postoperative Complications

There are some postoperative complications in ruptured globe injuries, such as a secondary cataract, corneal belted degeneration, iris atrophy, and an irregular pupil. Long-term contact between the silicone oil and lens is another factor for a secondary cataract. Fortunately, a cataract is easy to treat and correct.^22,23^ Cataract surgery was performed combined with intraocular lens implantation if the retina was in good condition and there was good visual acuity.^24,25^

Belted corneal degeneration is the result of limited secretion of aqueous humor. The poor circulation of aqueous humor results in concentrated calcium and the resulting belted corneal degeneration.^26,27^ Therefore, belted corneal degeneration indicates ciliary epithelium dysfunction and a precursor of eye atrophy.

Ocular trauma is a great challenge that can result in severe damage to visual function. We can try to restore some visual acuity, but there are still many aspects that need to improve such as treatment of choroidal trauma, proliferation pathology, and ciliary epithelium dysfunction. In addition, because our clinical sample is not large enough, some problems still need to be addressed. We hope that our experience can help junior oculists to improve their understanding of ruptured globe injuries.

## Conflicts of Interest

The authors have indicated that they have no conflicts of interest regarding the content of this article.

## Figures and Tables

**Table I:** Ruptured globe injury demographics data (N = 75).*

Demographic characteristics	
Male, n	64 (85.3)
Average age, y (range)	37.0 (6–63)
Eye	
Right	39 (52.0)
Left	36 (48.0)
Injury mechanism	
Accidental object (eg, wood, stone, chair)	62 (82.7)
Explosion	7 (9.3)
Fist	2 (2.7)
Traffic accident	3 (4.0)
Fall	1 (1.3)

⁎ Unless otherwise indicated, values are given as number (%).

**Table II:** Visual acuity after treatment in those with ciliary injury, choroidal injury, or both

Injury	Visual Acuity After Treatment				
	NLP	LP	≥HM, <FC	≥FC, <0.1	≥0.1
Ciliary	0	0	3	2	8
Choroidal	3	5	29	6	4
Ciliary and choroidal	9	4	2	0	0

FC = finger counting; HM = hand motion; LP = light perception; NLP = no light perception.
